# Correction: Andrews et al. Age at Arrival and Depression among Mexican Immigrant Women in Alabama: The Moderating Role of Culture. *Int. J. Environ. Res. Public Health* 2022, *19*, 5342

**DOI:** 10.3390/ijerph20042928

**Published:** 2023-02-08

**Authors:** Courtney Andrews, Kathryn S. Oths, William W. Dressler

**Affiliations:** 1 Institute for Human Rights, University of Alabama at Birmingham, Birmingham, AL 35294, USA; 2 Department of Anthropology, The University of Alabama, Tuscaloosa, AL 35487, USA

Several errors were introduced after proofreading, and the authors hence wish to make the following corrections to this paper [1]:

## Additional Affiliations

Kathryn S. Oths and WilliamW. Dressler are affiliated with the University of Alabama while Courtney Andrews is affiliated with the University of Alabama at Birmingham. They are both part of the UA system but are separate universities. The corrected Affiliations appear below.

^1^  Institute for Human Rights, University of Alabama at Birmingham, Birmingham, AL 35294, USA
^2^  Department of Anthropology, The University of Alabama, Tuscaloosa, AL 35487, USA

## Errors in Tables

In the original publication, there was a mistake in Table 2 as published. “Humilde” is missing a translation, which is “modesty.” The corrected Table 2 appears below.

In the original publication, there was a mistake in Table 3 as published. A clarifying definition is missing under Group 1 and Group 2. Underneath Group 1 should read, “priority: long-term goals”, and under Group 2 it should read, “priority: material goods”. The corrected Table 3 appears below.

In the original publication, there was a mistake in Table 4 as published. The values are incorrect. The corrected Table 4 appears below.

In the original publication, the decimals do not line up in Table 5. The decimals should line up all the way down the column, even when there is a negative sign in front of the number. Additionally, the *p*-values for Table 5 should be: Model 1—*p* = 0.10; Model 2—*p* = 0.10; Model 3—*p* = 0.07; Model 4—*p* = 0.01. The corrected Table 5 appears below.

## Text Correction

1. There was an error in the original publication. Section 5.1, paragraph 5: On the first use of the Spanish term “*familismo*”, please add in parenthesis the English translation: “A common sentiment expressed among older women was the change in family dynamics, specifically the weakening of a sense of *familismo* (familism).”

The corrected paragraph appears below.

A common sentiment expressed among older women was the change in family dynamics, specifically the weakening of a sense of *familismo* (familism). They expressed discomfort in not knowing their children’s friends or their parents, most often due to a language barrier. This posed a problem in communicating with their own children as well, as many had greater fluency in English and only spoke Spanish begrudgingly. This caused a profound sense of disconnect and discord in the homes—mothers frustrated with children for speaking to them in a language they did not understand, and children frustrated with mothers for not learning English. “They were young when they started with English. Me, I’m too old, and for me it just doesn’t stick”, one participant explained. The children’s proficiency in English did have its benefits, for example, if a mother needed a child to translate for her with a medical professional or other service agent. In another sense, however, this reversal of parenting roles was spoken of as a source of shame for the mothers and confusion for the children.

2. There was an error in the original publication. Section 7, paragraph 3: Buena (with a capital B) should be buena (with a lowercase b).

The corrected paragraph appears below.

We use a cultural models approach to better understand how participants conceptualize *la buena vida*. This approach can be distinguished from other work on prototypicality because it asks individuals to conceptualize an ideal, what they want their lives to be like, which may or may not align with their actual lives. The items mentioned in the free lists of what kinds of things are important or necessary to have *la buena vida* included material items indicative of a modern, middle-class lifestyle as well as character traits related to being a good person, particularly a good mother. This serves as a contrast to what Kaja Finkler describes as *la mala vida* (the bad life), which characterizes people (usually women) who are both economically and morally destitute [56]. It may be surprising to some readers that things such as safety, security and long-term immigration goals (i.e., gaining citizenship) were not mentioned often in the free lists. It seems that participants were thinking about *la buena vida* more in terms of what they can reasonably achieve or actualize in their everyday lives and less so in terms of governmental processes or policy changes that are necessary to ensure their citizenship or safety and protection under U.S. law. As mentioned in the qualitative themes, issues of insecurity and fear of deportation are central to the participants’ lives, so all of the results of the quantitative component of this study should be analyzed and understood in this overarching context.

The authors apologize for any inconvenience caused and state that the scientific conclusions are unaffected. This correction was approved by the Academic Editor. The original publication has also been updated.

## Figures and Tables

**Table 2:** Free-list terms for *la buena vida*.

Term	Frequency	Average Rank	Salience
Casa (House)	17	2.35	0.47
Tiempo con familia (Family time)	17	4.12	0.37
Coche (Car)	16	2.94	0.40
Positiva (Be positive)	13	6.77	0.14
Buen trabajo (Good job)	10	2.60	0.27
Dinero (Money)	9	4.33	0.19
Comida (Food)	8	5.13	0.15
Tiempo para estudiar (Study time)	6	6.17	0.09
Ropa (Clothes)	5	7.00	0.06
Aprender inglés (Learn English)	5	5.00	0.09
Educación para niños (Education for children)	5	7.6	0.05
Refrigerador (Refrigerator)	5	5.2	0.10
Internet (Internet)	4	3.00	0.09
Acceso a medicina (Affordable medicine)	4	9.75	0.02
Cuidado de salud (Health insurance)	4	6.75	0.05
Amigas (Friends)	4	6.50	0.05
Ayudar a otros (Help others)	4	6.50	0.04
Religiosa (Be religious)	4	4.50	0.08
Rezar (Pray)	3	9.67	0.02
Humilde (Modesty)	3	6.33	0.05
Horno/estufa (Oven/stove)	3	4.00	0.07
Tiempo libre (Free time)	3	5.67	0.06
Televisor (Television)	3	6.33	0.05
Ejercicio (Exercise)	3	6.67	0.03
Celular (Cell phone)	3	4.33	0.06
Ser amable (Be kind)	2	8.00	0.02
Ser espiritual (Be spiritual)	1	5.00	0.02

*n* = 1; Average response length = 8.74; Range = 5–13; Total items listed = 85.

**Table 3:** Cultural consensus analysis by subgroup (Sample 3).

	Group 1 (Priority: Long-Term Goals)	Group 2 (Priority: Material Items)
# of Respondents	27	14
Age	36.74 (22–54, 8.34)	32.07 (19–43, 6.78)
Age at Arrival	22.52 (9–48, 8.62)	17.77 (6–28, 6.31)
# of Years in U.S.	14.26 (6–29, 4.39)	13.14 (2–21, 4.93)
English Proficiency *	1.93 (1–3, 0.78)	2.86 (1–4, 1.03)
SES **	4.00 (2–6, 1.21)	5.31 (4–7, 1.12)
# of Negative Competence Scores	1	2
Average Competency (range, s.d.)	0.50 (−0.36–0.87, 0.26)	0.54 (−0.21–0.90, 0.36)
Eigenvalue ratio	3.06	3.01

* Self-assessed English proficiency reported as (0) none, (1) a little, (2) good, (3) very good; ** SES measured as average weekly salary of household (0–3) and highest education level completed (0–3); See Table 1.

**Table 4:** Bivariate correlation matrix between acculturation measures, cultural consonance, and depressive symptoms.

	Age at Arrival	Years in U.S.	English Proficiency	Depressive Symptoms	Cultural Consonance
Age at Arrival		−0.27 *	−0.24 *	0.25 *	−0.30 **
Years in U.S.			0.06	−0.09	−0.29
English Proficiency				−0.30 **	0.48 **
Depressive Symptoms					−0.30 *

* Significant at 0.05 level; ** Significant at 0.01 level.

**Table 5:** Regression models of age at arrival and cultural consonance on depressive symptoms.

	Model 1	Model 2	Model 3	Model 4
Age	0.19	0.07	0.11	0.21
SES	−0.16	−0.07	0.14	0.18
Years in U.S.	- - -	−0.07	−0.11	−0.22
English Proficiency	- - -	−0.22	−0.19	−0.21
Age at Arrival	- - -	0.13	0.04	−0.70
Cultural Consonance	- - -	- - -	−0.29	−0.22
Age at Arrival × Cultural Consonance	- - -	- - -	- - -	−0.31 *
	R^2^ = 0.04; *p* = 0.10	R^2^ = 0.06; *p* = 0.10	R^2^ = 0.08; *p* = 0.07	R^2^ = 0.15; *p* = 0.01

*N* = 70; All variables (except dependent) standardized; * = significant at 0.01 level.
